# Supplementary figures and images for: Developmental dynamics of sex reprogramming by high incubation temperatures in a dragon lizard
Source: BMC Genomics. 2022 Apr 22;23:322. doi: 10.1186/s12864-022-08544-2 (PMC9034607; doi:10.1186/s12864-022-08544-2)

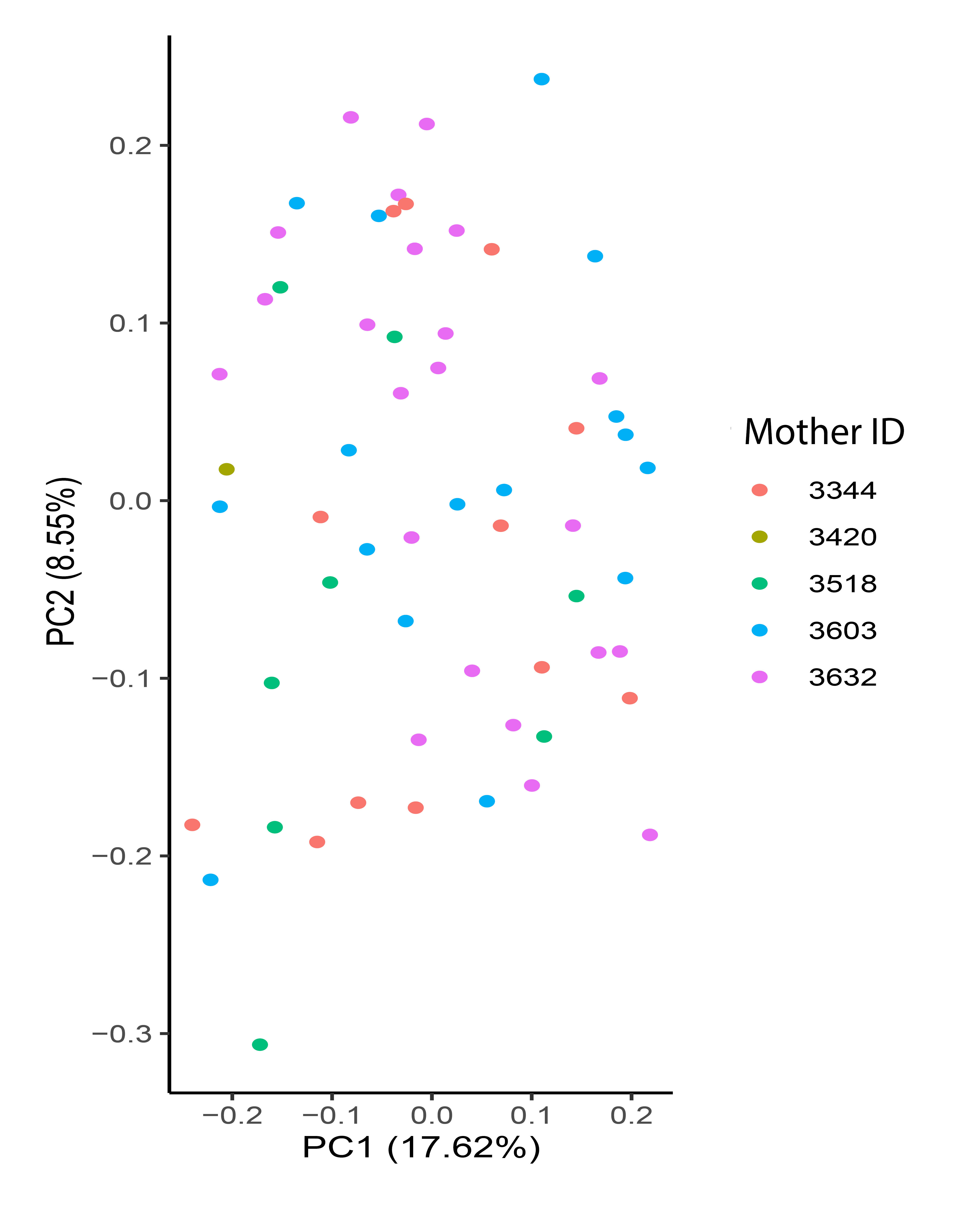

Supplement: Supplementary file 1 — Additional file 1: Figure S1. PCA plot for the complete dataset coloured by mother. The principal component analysis was conducted on normalised read counts. [file 12864_2022_8544_MOESM1_ESM.png]
